# 12 Weeks of Physical Exercise Attenuates Oxidative Stress, Improves Functional Tests Performance, and Reduces Fall Risk in Elderly Women Independently on Serum 25(OH)D Concentration

**DOI:** 10.3389/fphys.2022.809363

**Published:** 2022-04-20

**Authors:** Ewa Aleksandra Rodziewicz-Flis, Małgorzata Kawa, Damian Józef Flis, Marzena Szaro-Truchan, Wojciech Romuald Skrobot, Jan Jacek Kaczor

**Affiliations:** ^1^ Department of Basic Physiotherapy, Gdansk University of Physical Education and Sport, Gdansk, Poland; ^2^ Department of Pharmaceutical Pathophysiology, Medical University of Gdansk, Gdansk, Poland; ^3^ Department of Clinical Physiotherapy, Gdansk University of Physical Education and Sport, Gdansk, Poland; ^4^ Department of Animal and Human Physiology, University of Gdansk, Gdansk, Poland

**Keywords:** healthy ageing, fall risk, aerobic training, vitamin D, bone metabolism

## Abstract

The study aimed to evaluate if the 25(OH)D concentration is related to physical training responses. Moreover, to determine the association between serum 25(OH)D concentration and older women’s physical performance, oxidative stress markers, inflammation, and bone metabolism. 37 older women (age 72.9 ± 5.2 years) were assigned into two groups: supplemented (SG) and non-supplemented (NSG). Then, the participants from SG and NSG were randomly assigned into exercised and non-exercised groups: exercise sufficient vitamin D group (ESD; *n* = 10), exercise insufficient vitamin D group (EID; *n* = 9), control sufficient vitamin D group (CSD; *n* = 9), and control insufficient vitamin D group (CID; *n* = 9). To assess the study aims time up and go test (TUG), 6 min walk test (6MWT), fall risk test (FRT), blood osteocalcin (OC), parathormone (PTH), calcium (Ca^2+^), sulfhydryl groups (SH), malondialdehyde (MDA), and interleukin-6 (IL-6) were performed. The results showed that a higher 25(OH)D concentration was in line with better physical performance and bone metabolism as well as lower inflammation. After 12 weeks of training we noted an improvement in 6MWT (from 374.0 ± 17.3 to 415.0 ± 18.8; *p* = 0.001 and from 364.8 ± 32.8 to 419.4 ± 32.3; *p* = 0.001 for EID and ESD, respectively), TUG (from 7.9 ± 0.5 to 6.8 ± 0.8; *p* = 0.001 and from 7.3 ± 1.5 to 6.4 ± 0.9; *p* = 0.002, for EID and ESD, respectively), reduction of fall risk (from 2.8 ± 0.8 to 1.9 ± 0.4; *p* = 0.003 and from 2.1 ± 1.1 to 1.6 ± 0.5; *p* = 0.047, for EID and ESD, respectively) and increase in SH groups (from 0.53 ± 0.06 to 0.58 ± 0.08; *p* = 0.012 and from 0.54 ± 0.03 to 0.59 ± 0.04; *p* = 0.005, for EID and ESD, respectively), regardless of the baseline 25(OH)D concentration. A decrease in PTH and OC concentration was observed only in EID group (from 57.7 ± 15.7 to 49.4 ± 12.6; *p* = 0.013 for PTH and from 27.9 ± 17.2 to 18.0 ± 6.2; *p* = 0.004 for OC). To conclude, vitamin D concentration among older women is associated with physical performance, fall risk, inflammation, and bone metabolism markers. Moreover, 12 weeks of training improved physical performance and antioxidant protection, regardless of baseline vitamin D concentration.

## 1 Introduction

Aging is associated with many chronic diseases and pathological states, like sarcopenia, osteoporosis, chronic inflammation, and oxidative stress. In consequence, it may lead to disability, higher mortality, geriatric syndrome, and accelerated aging process (Campisi et al., 2019). One factor that may contribute to healthy aging is vitamin D status (Berridge, 2017). It is well known that vitamin D deficiency is a common health problem not only in the elderly. The prevalence of vitamin D deficiency is 50% or more among the senior population worldwide (van Schoor and Lips, 2011). The International Society for Clinical Densitometry and International Osteoporosis Foundation recommend a serum concentration of 25(OH)D at least 30 ng/ml to minimize the risk of fall and fractures in older individuals (Sizar et al., 2021). Serum 25(OH)D deficiency in the elderly may lead to increased inflammation, oxidative stress, muscle weakness, as well as more significant declines in physical performance as getting older (Bischoff-Ferrari et al., 2004; Wicherts et al., 2007; Berridge, 2017); however, the associations between vitamin D and physical performance remain controversial (Verreault et al., 2002; Annweiler et al., 2009).

The effect of vitamin D status on the risk of falls and physical performance may be related to several factors such as parathormone (PTH), bone turnover markers (BTM), cytokine concentration, and others. It is known that low vitamin D level leads to increased PTH concentration by impaired calcium metabolism. This phenomenon contributes to an increase in bone turnover and bone loss and is a risk factor for rickets and osteomalacia (Pludowski et al., 2013). Moreover, it has been shown that a higher level of PTH is related to increased risk of fracture and higher fall risk (de Franca et al., 2019). Consequently, maintaining an optimal vitamin D level may be essential to reduce fall risk and fractures. Osteocalcin (OC) is one of the BTM also related to vitamin D status, and it is produced by bone osteoblasts and partly released into the bloodstream (Schwetz et al., 2012). A previous study indicates that serum OC concentration decreased while serum mean 25(OH)D increased (Kuchuk et al., 2009). Moreover, a human with osteoporosis and osteopenia has been shown to have elevated serum OC concentration (Wang et al., 2019).

Furthermore, it has been proposed that vitamin D may affect aging processes also by controlling the activity of several cellular processes, among others oxidative stress or inflammation. These are one of the main drivers of aging and can be enhanced in individuals with vitamin D deficiency (Berridge, 2017). Insufficient serum vitamin D concentration may be related to increased cell damage induced by reactive oxygen species (ROS) through its ability to control the expression of cellular antioxidants (Dzik and Kaczor, 2019). Sufficient vitamin D concentration reduces inflammation by decreasing pro-inflammatory cytokines (Krasowska et al., 2019).

One of the well-known markers of chronic systemic inflammation in older adults is an interleukin-6 (IL-6) (Maggio et al., 2006). The circulating concentration of IL-6 higher than 2.5 pg/ml is a cut-off point indicating low-grade inflammation and a higher risk of functional decline (Ferrucci et al., 1999). Moreover, higher IL-6 concentration negatively affects bone metabolism by stimulating osteoblasts precursors differentiation and osteoclasts formation *via* an increase in RANKL expression by osteoblasts. Thus, it may be associated with the pathogenesis of postmenopausal osteoporosis; however, this data remains controversial (Kaji, 2016). On the other hand, it has also been suggested that IL-6 might be related to physical performance in older adults, but these data are both limited and inconsistent (Custodero et al., 2020; Grosicki et al., 2020).

Besides vitamin D status, physical activity also contributes to healthy aging by reducing the risk of chronic diseases, slowing aging-related processes, or preventing loss of independence (Campisi et al., 2019). The positive impact of exercise on age-related chronic diseases and physical performance may be associated with modifying both muscle and bone-derived proteins or hormones concentrations (Safdar and Tarnopolsky, 2018). Systemic humoral factors produced from muscle or bone tissue in response to exercise affect each other and may mediate the positive impact of physical exercise (Kaji, 2016). Furthermore, physical activity is known for its anti-inflammatory and antioxidant effects (Bachi et al., 2019). Nevertheless, little is known if vitamin D insufficiency may regulate training responses and training adaptations. It has been previously indicated that vitamin D status is related to physical performance only at baseline; however, its supplementation in addition to physical training did not enhance training responses. Previous studies indicated that vitamin D supplementation before and during the resistance training program did not show additive effects compared to physical training applied alone in male and female elders (Antoniak and Greig, 2017; Molmen et al., 2021). On the other hand, it has been suggested that responses to resistance training, especially muscle mass gains, are related not to blood vitamin D concentration but enhanced vitamin D receptor expression in skeletal muscle (Bass et al., 2020).

The first purpose of this study was to determine if the baseline concentration of 25(OH)D is related to physical training responses. The second was to estimate the association between serum 25(OH)D concentration and older women’s physical performance, fall risk, oxidative stress markers, inflammation, and bone metabolism. We hypothesized that regular physical activity decreases oxidative stress and inflammation markers, improves functional tests performance, and reduces fall risk in elderly women independently on serum 25(OH)D concentration.

## 2 Materials and Methods

### 2.1 Participants

**FIGURE 1:**
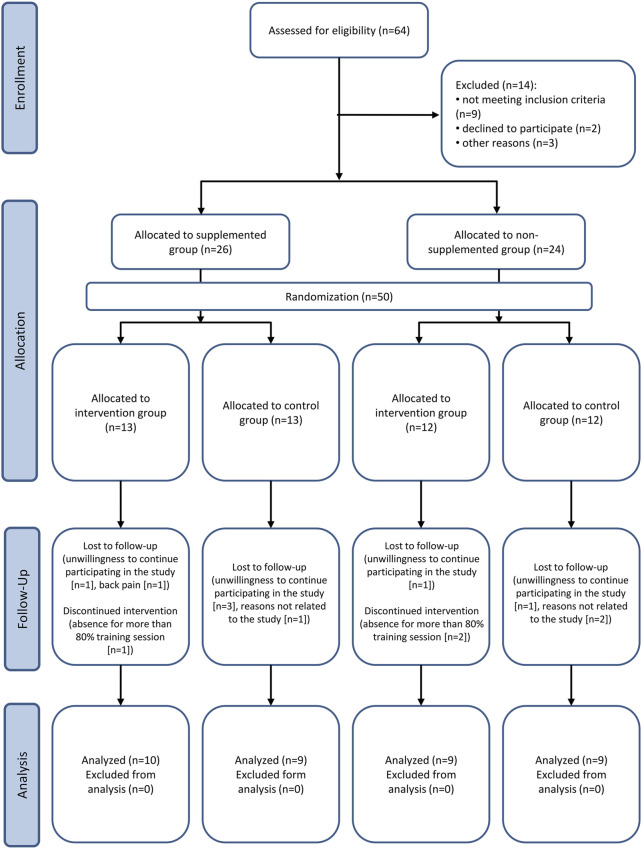
CONSORT flow diagram of the study.

Fifty community-dwelling female participants (age 72.9 ± 5.2 years, range 65–82) were enrolled in the study (Figure 1). Participants were recruited from advertisements in community centers in Gdansk, Poland. All participants were lived and functioned independently, in good health, without severe cognitive or physical impairments. The inclusion criteria for the study were: female, age of 65 or older, and low to moderate physical activity assessed by International Physical Activity Questionnaire—IPAQ (short version). This questionnaire measures the recreational activity, housework, and physical activity performed over the past years. Before the study, participants also completed the questionnaire regarding medications and supplements taken, including vitamin D. The exclusion criteria were: any diseases accompanied by contraindication to exercise and required specialized treatment, coronary disease, arrhythmia, implanted pacemaker, heart failure, depression, cancer, significant orthopedic injuries, disabling dyspnea, taking medications for depression and heart diseases. We also excluded participants who regularly participated in exercise programs such as strength training, balance training, or aerobic training for 12 months before the study. Of the 50 participants included, 13 withdrew from the study during the follow-up. Finally, thirty-seven older women completed the study (Figure 1). All women were informed about the research’s risks and purpose and familiarized with the study methods. The study was approved by the local institutional Bioethical Committee in Gdansk (NKBBN/455/2018) and conformed to the Declaration of Helsinki. Written informed consent was obtained from the participants of the study. The trial was registered at ClinicalTrials.gov (Identifier: NCT03417700).

### 2.2 Study Design

The study was a single-blind, randomized trial. All assessments (body composition analysis, functional tests, fall risk test, blood analysis) at baseline and after training intervention were performed by researchers blinded to the group allocation. At first, based on participants answer to the question: whether they use vitamin D supplements of 2,000 units or more, two groups were performed: supplemented with vitamin D (SG); *n* = 19 or not (NSG); *n* = 18 in order to evaluate if there are differences in physical performance, fall risk, inflammation, oxidative stress, and bone metabolism among groups at baseline of the experiment. The criterion that qualified a person to the supplemented group was taking 2,000 units or more of vitamin D per day for at least 2 months. Additionally, after the baseline assessments, participants from SG and NSG were randomly assigned into exercised and non-exercised groups: exercise sufficient vitamin D group (ESD; *n* = 10), exercise insufficient vitamin D group (EID; *n* = 9), control sufficient vitamin D group (CSD; *n* = 9), and control insufficient vitamin D group (CID; *n* = 9). The ESD and EID performed training three times a week (Monday, Wednesday, Friday) for 12 weeks. The training procedure started 2 days after the baseline measurements (blood collection, body composition analysis, 6-min walk (6MWT), time up and go (TUG), and fall risk (FRT) tests). Also, 2 days after the last training session, all of the measurements mentioned above were repeated. The blood was collected from the participants to assess OC, PTH, 25(OH)D, IL-6, sulfhydryl (SH) groups, malondialdehyde (MDA), and calcium (Ca^2+^) concentration. Participants were asked not to change their daily habits during the experiment, including supplementation.

### 2.3 Intervention

#### 2.3.1 Experimental Group

Exercise groups completed 12 weeks of combined aerobic-resistance training with moderate intensity, supervised by a physiotherapist. Participants trained 3 days a week for 50 min per session (150 min of activity per week), totaling 36 training sessions. The training was conducted on Mondays, Thursdays, and Fridays. Participants performed aerobic exercises combined with resistance exercises. The exercise intensity was prescribed at 60% of maximum heart rate during the first 4 weeks, then gradually increased to 80% of HRmax during the last 4 weeks. The heart rate was measured by pulsometer (Polar Heart Rate Monitor, Polar Electro, Finland) and was between 100–140 beats per minute during the training sessions. Each training session consisted of 5 min of warm-up, 20 min of aerobic exercise, 20 min of resistance exercise, and 5 min of cool-down. The aerobic training periods contained standard fitness and balance exercises performed to the music. During the resistance part of the training, participants performed exercises with their body weight and with external load (dumbbells, resistance band) for upper and lower limbs and abdominal muscles. Exercises were performed with a 2–5 kg load and were designed to improve strength endurance. Participants performed about 12–25 repetitions of each exercise with a 30–60 s break. Resistance training intensity increased every 4 weeks (12–15 repetitions in weeks 1–4, 15–20 repetitions in weeks 5–8, and 20–25 repetitions in weeks 9–12). The exercises used in resistance training were, for example, as follows: knee push-ups, squats, forward lunges, back lunges, crunches, standing dumbbell press, dumbbell lateral raise, dumbbell bench press, toe lifts. The muscles trained in the resistance training periods were: biceps brachii, triceps brachii, deltoid, pectoralis, abdominal muscles, back muscles, gluteus muscles, and lower limbs muscles. The minimal training attendance was 80%, and the mean training attendance was 89%.

#### 2.3.2 Control Group

The control group did not participate in any training intervention. Participants were instructed not to change their daily habits and maintain current activity during the 12-weeks study period.

### 2.4 Measurements

#### 2.4.1 Body Composition Analysis

Body composition analysis was estimated by a precise multi-frequency impedance plethysmography body composition analyzer (InBody 720, Biospace, Korea), using six different frequencies (1 kHz, 5 kHz, 50 kHz, 250 kHz, 500 kHz, and 1000 kHz) at each of five segments of the body (Right Arm, Left Arm, Trunk, Right Leg, Left Leg). The measurement was performed with the 8-point tactile electrode method. InBody measures body composition by segment, and test duration is approximately 60 s. Parameters as body weight, free fat mass (FFM), body fat mass (FM), skeletal muscle mass (SMM), and visceral fat (VFA) have been assessed. The body composition analysis was performed by one researcher for all participants in a fasted state, 12 h after the last meal and drink, between 9:00–10:00 in the morning after blood collection, at baseline (2 days before first training session) and 2 days after the last training session. Before contacting with electrodes, participants disinfected their hands and feet with alcohol. During the assessment, women were barefoot, dressed only in underwear and without jewelry, watches, etc. The impedance measurements were made standing in an upright position, with feet and hands centered on the electrodes, and the upper limbs positioned around 30° away from the trunk. This position was held for the test duration (McLester et al., 2020).

#### 2.4.2 Blood Analysis and Collection

Blood samples for 25(OH)D, OC, PTH, Ca^2+^, SH groups, MDA, and IL-6 assessment were collected between 8:00–9:00 after overnight fasting at two time-points: at baseline (2 days before the intervention) and 2 days after completion the 12-weeks training procedure. A qualified nurse took venous blood from the antecubital vein into vacutainer tubes (Vacutainer SSTTM II Advance) for serum separations and tubes with EDTA as an anticoagulant for plasma isolation. The samples were centrifuged at 2000 × g for 10 min at 4°C. The separated plasma and serum samples were frozen and stored at −80°C until later analysis. Plasma OC and PTH (hormone regulating calcium metabolism) concentrations were determined using the immunoenzymatic (ELISA) method using commercially available kits (R&D, Unites States & Canada, No. DSTCN0 and Demeditec diagnostics, Germany, No. DE3645, respectively) according to the manufacturer’s protocol. The maximal intra-assay coefficient of variability (CV) was 1.6%–3.4%, and inter-assay CV was 6.1%–6.9% for OC and 3.68%–6.08% and 2.8%–3.6% for PTH. The assay sensitivity was 0.898 ng/ml for OC and 0.761 pg/ml for PTH. IL-6 was assessed in serum also by immunoenzymatic method using ELISA Kit - R&D, United States & Canada, No. HS600B). The maximal intra-assay and inter-assay CV for IL-6 was 6.9%–7.4% and 6.5%–9.6%, and the assay sensitivity was 0.11 pg/ml. Ca^2+^ concentration was assessed by plate-based colorimetric method (Cayman chemical, United States, No. 701220). The maximal intra-assay and inter-assay CV for Ca^2+^ was 3% and 5.96%, and the assay sensitivity was 0.25 mg/dl. Serum 25(OH)D was assessed by ELISA Kit (Demeditec diagnostics, Germany, No. DE1971) according to the manufacturer’s protocol. The maximal intra-assay CV was 2.5%–7.8%, and inter-assay was 7.4%–9.2%. The assay sensitivity was 2.81 ng/ml. The concentration of 25(OH)D below 30 ng/ml was classified as insufficient.

#### 2.4.3 Manifestation of Oxidative Stress

Plasma SH groups (a marker of protein peroxidation) were measured spectrophotometrically according to Kaszubowska and coworkers (Kaszubowska et al., 2011) against the standard curve (standard–reduced glutathione). The lowest concentration of reduced glutathione in the standard curve was 0.25 mmol/L. The range of the standard curve was between 0 and 2 mmol/L. The MDA concentration (a marker of lipid peroxidation) was measured spectrophotometrically using the LPO-586 assay (OxisReserch, Portland, United States) according to the manufacturer’s instructions, against the standard curve (standard–1,1,3,3 Tetramethoxypropane). The lowest concentration of 1,1,3,3 Tetramethoxypropane in the standard curve was 2.5 μmol/L. The range of the standard curve was between 0 and 150 μmol/L. The SH groups and MDA values were expressed as mmol/L and as µmol/L, respectively.

#### 2.4.4 Time Up and Go Test (TUG) and 6 min Walk Test (6MWT)

The TUG test is used to assess functional balance, mobility, and indirectly also a risk of falls. The test time of 13.5 s or above is associated with a two- to three-fold higher risk of falls (Shumway-Cook et al., 2000). All women were provided with standardized verbal instructions before the test started. Participants were asked to sit on a back-supported chair with arms crossed on the chest. Then, participants were instructed to get up from the chair on command, walk 3 m, turn in a signed place, walk back and sit down again. The time taken to perform the task was measured by a stopwatch by a qualified physiotherapist. Every participant performed the test twice, first slow with the researcher’s assistance to familiarize herself with the procedure. Then, after 5 min of rest, independently, as fast as possible, without running.

6MWT assessed the walking distance of patients within 6 min. The test result could indicate the functional status and elders’ respiratory, cardiovascular, and locomotor systems (Baddini-Martinez, 2018). The 6MWT was performed in a 30 m hallway. Women were asked to begin walking at a command “start” and walk back and forth as much as possible in 6 min with no running. The distance was measured by the measuring wheel. One researcher tested each participant. The TUG and 6MWT tests were separated by 10 min of rest.

#### 2.4.5 Fall Risk Test (FRT)

To evaluate the fall risk, participants received baseline and post-treatment assessments using the Biodex Balance System. The FRT is designed to identify potential fallers. This test protocol gives the age-adjusted normative data to assess the patient’s risk of falling. During the test, participants performed three trials of 20 s. Each trial began with an initial platform setting of 6 and ended with 2. Before the first test trial, the screen provided 3 s countdown. Between each trial, participants had 10 s rest. During the rest period, the platform returned to the locked, stable position. In order to perform the test, patients should stand in a comfortable position on the platform bilaterally with feet shoulder-width apart over the midline of the platform. Three test trials were used to avoid excessive balance deviations. Participants were asked to stand without support, look straight and focus on the visual feedback screen. After the test, the results are shown on the screen and compared to the normative data. The patients’ performance is noted as a stability index (SI). Scores higher than normative age values suggest a higher fall risk, indicating balance impairments. Lowering SI after the training program indicates balance control improvement.

### 2.5 Statistical Analysis

Statistical analyses were performed using the Statistica v.13.software package (StatSoft Inc., Tulsa, OK, United States). The participants’ characteristic were analyzed using descriptive statistics and is presented as mean ± standard deviation (SD). The Shapiro-Wilk test was determined for parameters distribution normality. For the baseline analysis the Mann-Whitney U (M-WU) test was used if the distribution was abnormal (for age, IL-6, SH, MDA, OC, Ca, 6MWT). If the parameters presented normal distribution, equality of group variances *via* the Brown-Forsyth test was verified. All groups presented equal variances; therefore, the unpaired student t-test was used for data with normal distribution. The differences between values before and after the training program were tested using the two-way repeated measurements of ANOVA. The Fisher’s Least Significant Difference (LSD) post-hoc test determined significant differences if a difference was detected in the ANOVA model. A Pearson product-moment correlation coefficient was computed to assess the relationship between the obtained results. Multivariate regression analysis was carried out to recognize the strength of each parameter predicting functional tests performance, fall risk, and IL-6 concentration. The results were considered statistically significant for *p* values less than 0.05.

## 3 Results

### 3.1 Baseline Characteristic of Participants From Supplemented and Non-Supplemented Group

The baseline group’s characteristic is presented in Table 1. None of the participants took osteoporosis drugs such as bisphosphonates before or during the study. All women had low to moderate daily physical activity levels measured by The Physical Activity Questionnaire–IPAQ (short version). The mean age of the participants from the supplemented group was 72.9 ± 5.1, and the non-supplemented group was 72.8 ± 5.3 years. We observed significant differences between groups in 25(OH)D concentration. The supplemented group’s 25(OH)D serum concentration was 43.2 ± 10.2 ng/ml, and in the non-supplemented group, the mean concentration was 16.9 ± 7.7 ng/ml (*p* = 0.001). The SG had significantly lower body mass, BFM, and VFA when compared to NSG (*p* = 0.015; *p* = 0.026 and *p* = 0.033, respectively). Moreover, the SG had a substantially lower PTH plasma concentration than the NSG (*p* = 0.001) at baseline. Before the intervention, no significant differences had been found in OC concentration. At baseline, the group with higher serum 25(OH)D concentration tends to have lower inflammation marker–IL-6 (*p* = 0.080 (M-WU); cohen’s d = 0.72). No differences were detected in oxidative stress markers between groups. Interestingly, significant differences were observed between SG and NSG groups in physical performance. Before the training procedure, the SG was characterized by better results in TUG (*p* = 0.032), lower fall risk (*p* = 0.030), and tend to have better results in 6MWT (*p* = 0.071; cohen’s d = 0.61).

**TABLE 1:** Characteristics of the participants.

Variables	NSG (*n* = 19)	SG (*n* = 18)	*P* value	Cohen’s d	Mean differences
Age (years)	72.8 ± 5.3	72.9 ± 5.1	ns	0.02	0.12
Height (cm)	1.61 ± 0.04	1.59 ± 0.04	ns	0.40	0.02
Weight (kg)	73.4 ± 8.1	66.5 ± 8.2*	0.015	**0.88**	7.17
BMI (kg/m^2^)	27.9 ± 3.4	26.0 ± 3.1	ns	**0.59**	2.21
FFM (kg)	45.9 ± 4.7	43.5 ± 4.4	ns	**0.51**	2.34
BFM (kg)	29.0 ± 7.7	23.9 ± 5.5*	0.026	**0.76**	5.13
SMM (kg)	24.9 ± 2.7	23.6 ± 2.6	ns	0.48	1.28
VFA (cm^2^)	115.9 ± 28.4	98.4 ± 21.1*	0.033	**0.61**	15.31
Vitamin D (ng/ml)	16.9 ± 7.7	43.2 ± 10.2*	0.001	**2.92**	26.32
PTH (pg/ml)	59.1 ± 16.6	37.2 ± 9.7*	0.001	**1.61**	21.91
OC (ng/ml)	29.7 ± 20.7	22.1 ± 10.0	ns	0.47	7.57
IL-6 (pg/ml)	2.96 ± 1.8	1.96 ± 0.8*	ns	**0.72**	1.01
Ca (mg/dl)	12.1 ± 1.5	12.5 ± 1.8	ns	0.22	0.36
SH (mmol/L)	0.57 ± 0.09	0.56 ± 0.05	ns	0.12	0.01
MDA (µmol/L)	0.079 ± 0.03	0.069 ± 0.03	ns	0.37	0.01
TUG (s)	8.48 ± 1.3	7.50 ± 1.4*	0.032	**0.74**	0.98
6MWT (m)	333.8 ± 60.9	364.7 ± 38.2	ns	**0.61**	30.96
Fall risk	2.89 ± 0.8	2.14 ± 1.2*	0.030	**0.75**	0.75

Values are given as mean ± SD. Ns, non-significant differences between groups; Cohen’s d, the Cohen’s effect size [effect sizes as small (d = 0.2), medium (d = 0.5), large (d = 0.8), and very large (d = 1.3), moderate to very large effect size have been highlighted in bold]; mean differences, mean differences between SG and NSG group. NSG, non-supplemented group; SG, supplemented group; BMI, body mass index; FFM, free fat mass; BFM, body fat mass; SMM, skeletal muscle mass; VFA, visceral fat area; PTH, parathormone; OC, osteocalcin; IL-6, interleukin-6; Ca, calcium; SH, sulfhydryl groups; MDA, malondialdehyde; TUG, time up and go test; 6MWT, 6 min walk test.

*p < 0.05, a significant difference between supplemented and non-supplemented group at baseline.

### 3.2 Pearson’s Correlations of 25(OH)D, a Marker of Inflammation, Bone Metabolism Markers, and Physical Performance

The negative correlation between vitamin D and body mass was found (r = −0.33; *p* = 0.003). We also found a significant negative correlation between 25(OH)D and IL-6 among all study groups (r = −0.34; *p* = 0.002). Moreover, the higher 25(OH)D concentration was associated with lower PTH concentration (r = −0.59; *p* = 0.001) and lower OC (r = −0.28; *p* = 0.01). A significant association between 25(OH)D concentration and physical performance was also observed. A negative correlation was noted between 25(OH)D and TUG test time (r = −0.46; *p* = 0.001) as well as fall risk (r = −0.26; *p* = 0.017). Furthermore, 25(OH)D concentration positively correlates with the distance performed during 6MWT (r = 0.33; *p* = 0.003). The association between inflammation and body composition as well as physical performance was also observed. We noted the positive correlation between IL-6 concentration and TUG and BFM (r = 0.45; *p* = 0.001; r = 0.29; *p* = 0.008, respectively) and a negative correlation between IL-6 and 6MWT (r = −0.34; *p* = 0.002).

### 3.3 Multivariate Regression Analysis

Multivariate regression analyses were performed to investigate factors measured in the blood associated with functional test performance, fall risk, and IL-6 concentration. Multivariate analysis for TUG as a dependent variable showed that concentration of 25(OH)D and IL-6 are strong predictors (β = −1.171, *p* = 0.006 for 25(OH)D and β = 1.069, *p* = 0.001 for IL-6). Multivariate analysis for 6MWT as a dependent variable showed that concentration of 25(OH)D and IL-6 are strong predictors (β = 1.473, *p* = 0.001 for 25(OH)D and β = −0.933, *p* = 0.003 for IL-6). No significant associations were observed for FR as a dependent variable; however, 25(OH)D and OC pretend to be predictors (β = −0.864, *p* = 0.074 for 25(OH)D and β = −0.772, *p* = 0.063 for OC). Multivariate analysis for IL-6 as a dependent variable showed that 25(OH)D concentration, as well as BMI are a strong predictors (β = −1.305, *p* = 0.005 for 25(OH)D and β = −3.769, *p* = 0.047 for BMI).

### 3.4 Post-Training Changes in Physical Performance and Fall Risk

**FIGURE 2:**
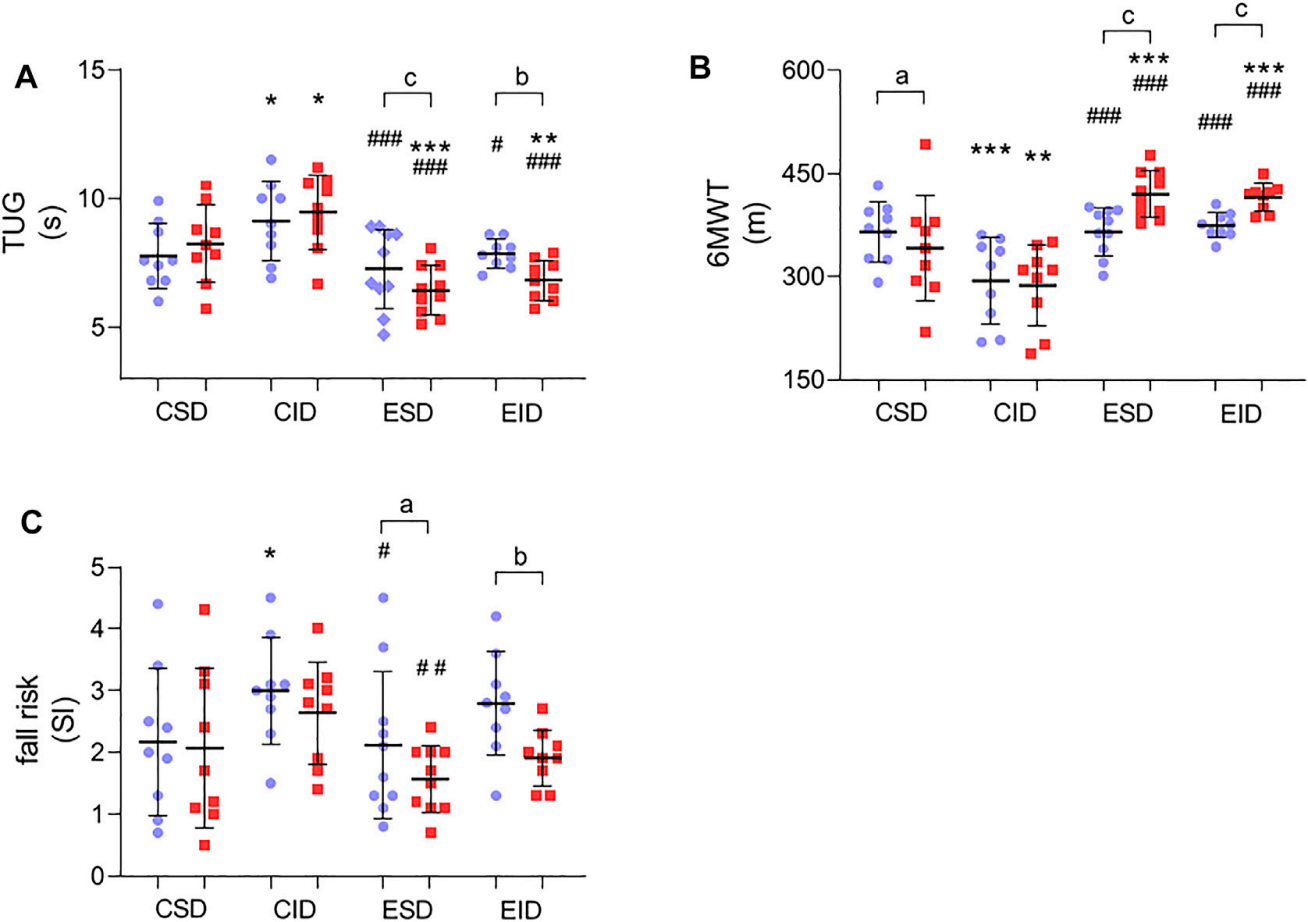
Post-training changes in physical performance and fall risk. **(A)** Time up and go test; **(B)** 6-minute walk test; **(C)** fall risk test. There were significant differences between the indicated time points: **a** – *p* < 0.05, **b** – *p* < 0.01, **c** – *p* < 0.001; between the groups: *p < 0.05, **p < 0.01, ***p < 0.001 vs. CSD; #*p* < 0.05, ##*p* < 0.01; ###*p* < 0.001 vs. CID. The data are presented as the means ± SEM; plain—before the intervention; strips—after the intervention.

After 12 weeks of a training program, we found a significant improvement in physical performance (Figure 2). For the TUG, there was a non-significant effect for time [F_(1,37)_ = 3.866, *p* = 0.057, η2 = 0.095], but a significant group × time interaction [F_(3,37)_ = 8.741, *p* = 0.0002, η2 = 0.415. A Fishers LSD post-hoc test showed a significant amelioration in TUG test time in both exercise groups (*p* = 0.002 for ESD and *p* = 0.001 for EID) (Figure 2A). For the 6MWT, there was a main effect for time [F_(1,37)_ = 8.734, *p* = 0.005, η2 = 0.191] and a significant group × time interaction [F_(3,37)_ = 11.748, *p* = 0.00001, η2 = 0.488] (Figure 2A). A used post-hoc test revealed an increase in the performed distance during the 6MWT in the ESD group (*p* = 0.001) and EID group (*p* = 0.001). Moreover, the decrease in the performed distance was found in the CSD group after 12 weeks of the experiment (*p* = 0.039) (Figure 2B). For the fall risk, there was a main effect for time [F_(1,37)_ = 11.295, *p* = 0.0018, η2 = 0.234] but a non-significant group × time interaction [F_(3,37)_ = 1.339, *p* = 0.277, η2 = 0.098]. The lower FR was found in the EID (*p* = 0.003) and ESD (*p* = 0.047) in Fishers LSD post-hoc test (Figure 2C). No changes were observed in the control groups.

### 3.5 Post-Training Changes in Bone Metabolism Markers and PTH

**FIGURE 3:**
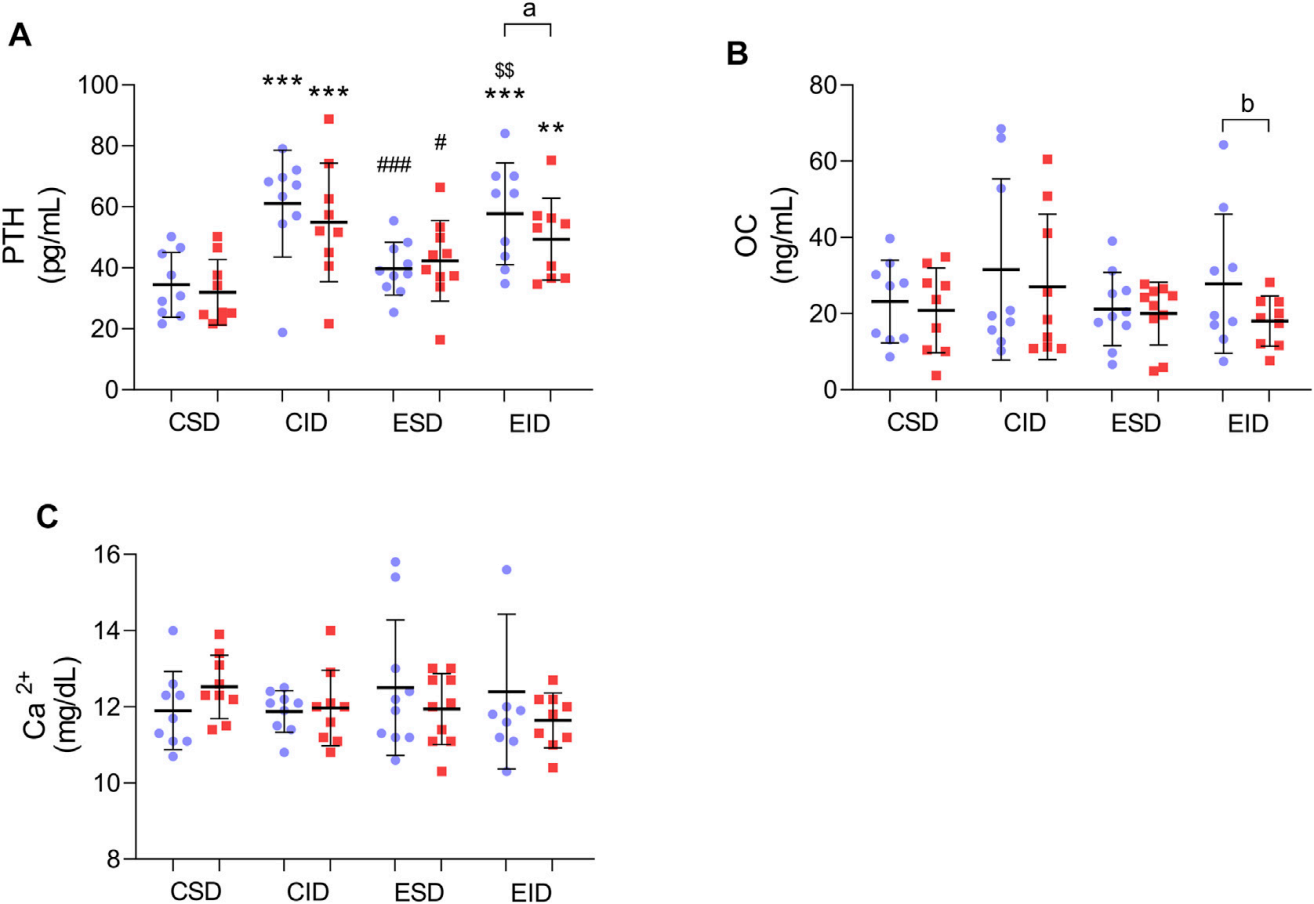
Post-training changes in bone metabolism and PTH. **(A)** PTH concentration; **(B)** OC concentration; **(C)** Ca^2+^ concentration. There were significant differences between the indicated time points: **a** – *p* < 0.05, **b** – *p* < 0.01; between the groups: **p < 0.01, ***p < 0.001 vs. CSD; #*p* < 0.05, ###*p* < 0.001 vs. CID; $$*p* < 0.01 vs. ESD. The data are presented as the means ± SEM; plain—before the intervention; strips—after the intervention.

For the PTH, there was no main effect for time [F_(1,37)_ = 3.859, *p* = 0.057, η2 = 0.094] and for a group × time interaction [F_(3,37)_ = 1.729, *p* = 0.178, η2 = 0.123]. Nevertheless, a Fishers LSD post-hoc test revealed a significant reduction in PTH concentration after 12 weeks of training, but only in the exercise group with insufficient 25(OH)D concentration (*p* = 0.031). In the ESD group, we did not find any significant changes (Figure 3A). For the OC, there was a main effect for time [F_(1,37)_ = 7.776, *p* = 0.008, η2 = 0.174] but no effect for a group × time interaction [F_(3,37)_ = 1.429, *p* = 0.250, η2 = 0.104]. An alteration in OC concentration was shown by a post-hoc test. Its reduction was noted only in the EID group (*p* = 0.004) (Figure 3B). For the calcium, there was neither effect for time [F_(1,37)_ = 0.418, *p* = 0.522, η2 = 0.011], nor for a group × time interaction [F_(3,37)_ = 2.017, *p* = 0.128, η2 = 0.141]. A slight but insignificant reduction in Ca^2+^ concentration was observed among both groups (Figure 3C).

### 3.6 Post-Training Changes in Inflammation Marker and Oxidative Stress

**FIGURE 4:**
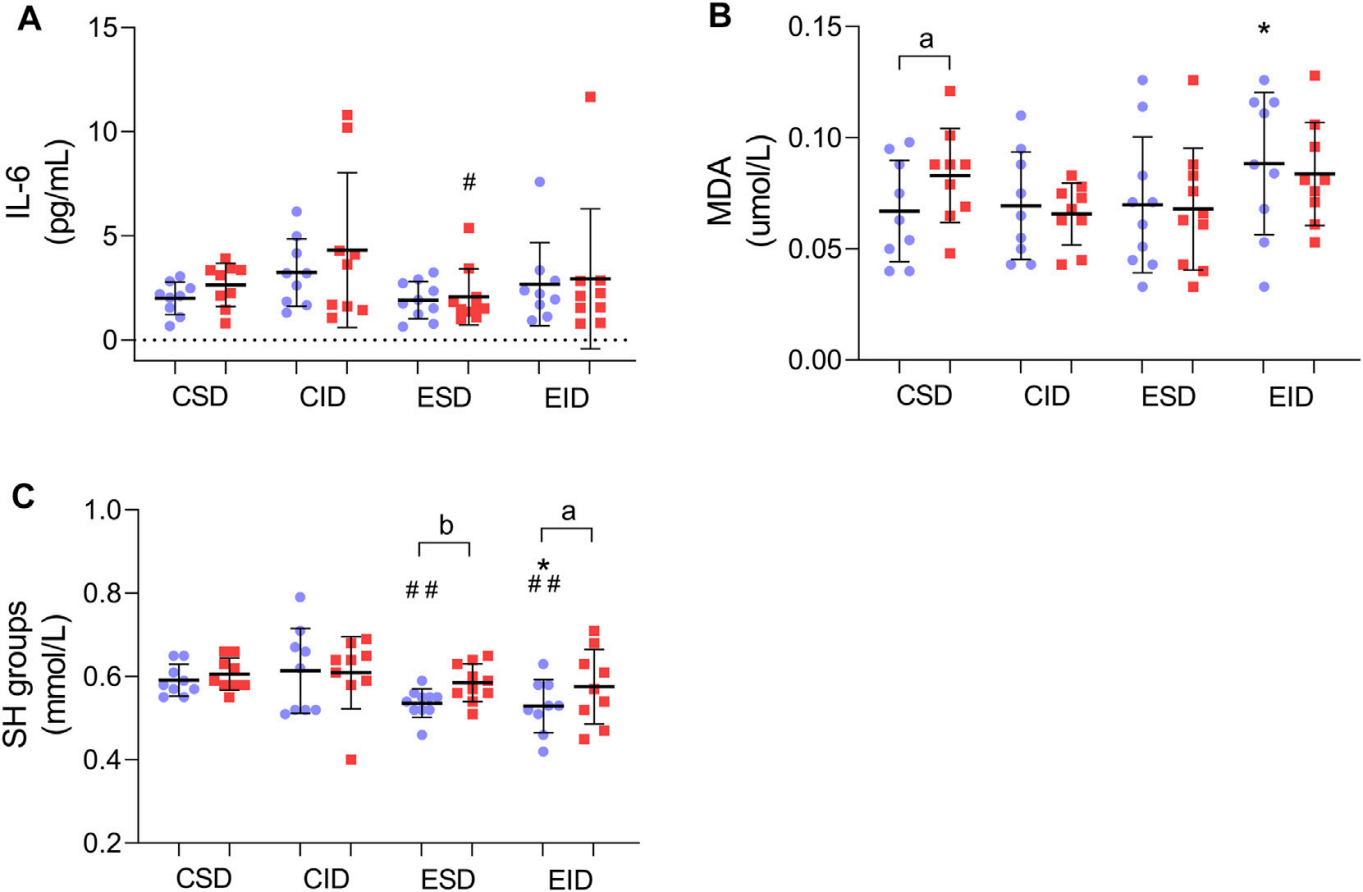
Post-training changes in inflammation marker and oxidative stress. **(A)** IL-6 concentration; **(B)** MDA concentration; **(C)** SH concentration. There were significant differences between the indicated time points: **a** – *p* < 0.05, **b** – *p* < 0.01; between the groups: *p < 0.05 vs. CSD; #*p* < 0.05, ##*p* < 0.01 vs. CID. The data are presented as the means ± SEM; plain—before the intervention; strips—after the intervention.

We did not distinguish any significant changes in IL-6 concentration after the training program (Figure 4A). For the IL-6, there was an insignificant effect for time [F_(1,37)_ = 2.750, *p* = 0.106, η2 = 0.069] and for a group × time interaction [F_(3,37)_ = 0.399, *p* = 0.754, η2 = 0.031]. The 12-weeks of training also did not change the MDA concentration in any of the training groups. For the MDA, there was neither effect for time [F_(1,37)_ = 0.155, *p* = 0.696, η2 = 0.004] nor a group × time interaction [F_(3,37)_ = 1.752, *p* = 0.173, η2 = 0.124] (Figure 4B). For the SH groups, there was a main effect for time [F_(1,37)_ = 10.243, *p* = 0.003, η2 = 0.217] and an insignificant group × time interaction [F_(3,37)_ = 2.154, *p* = 0.110, η2 = 0.149]. The Fishers LSD test revealed an increase in SH groups concentration in both exercised participants–EID (*p* = 0.012) and ESD (*p* = 0.005) (Figure 4C).

## 4 Discussion

To our knowledge, for the first time, this research assessed if, among healthy, older women, the physical training responses are related to baseline vitamin D concentration. Our study confirmed an association between older women’s serum 25(OH)D concentration, a marker of inflammation, and physical performance. Moreover, this study pointed out the positive impact of moderate-intensity regular training on older women’s physical performance, fall risk, and oxidative stress regardless of baseline 25(OH)D concentration, indicating the physical activity as the most crucial factor leading to healthy aging.

The association of vitamin D concentration with older adults’ physical performance remains controversial and conflicting. The research in recent years showed an association between vitamin D deficiency and sarcopenia, increased fall risk, impaired muscle strength, and physical performance (Dhaliwal and Aloia, 2017; Remelli et al., 2019). The highest prevalence of lower muscle strength and physical performance decline was noted among older adults with a serum 25(OH)D concentration below 10 ng/ml (Aspell et al., 2019). Our study confirmed a correlation between physical performance and vitamin D concentration. Women with a 25(OH)D concentration higher than 30 ng/ml performed significantly better in the TUG and fall risk test and tended to have better results in 6MWT, indicating vitamin D status as a significant factor related to poor physical performance and higher fall risk in the elderly. Moreover, multivariate regression analysis indicated vitamin D concentration as one of the most important predictors of functional test performance and predicts fall risk. Therefore, vitamin D may be a potentially modifiable factor associated with maintaining physical performance and preserving independence as getting older. It is also known that vitamin D insufficiency leads to increased inflammation and intensified production of pro-inflammatory cytokines (Berridge, 2017). Our study is in line with these observations. We found a significant negative correlation between 25(OH)D and IL-6–a marker of systemic inflammation. The participants with 25(OH)D concentrations below 30 ng/ml had IL-6 concentrations higher than 2.5 pg/ml, a cut-off point indicating low-grade inflammation (Ferrucci et al., 1999). The multivariate regression analysis performed in the current study revealed that an insufficient 25(OH)D concentration strongly predicts inflammation development. Although the effect of vitamin D on muscle tissue is well known, the mechanism by which vitamin D acts positively on physical exercise is not fully understood. One of the potential mechanism(s) might be associated with suppressed production of pro-inflammatory cytokines while the vitamin D concentration is sufficient.

The higher concentration of IL-6 in the elderly is also suggested to induce physical performance decline, especially when combined with insufficient vitamin D concentration (Kositsawat et al., 2020). A negative correlation between IL-6 and functional test results was also observed. Therefore, a higher level of inflammation marker is associated with worse physical performance. The multivariate regression analysis confirmed that IL-6 and 25(OH)D concentrations are the strongest predictors of functional test performance.

The association of vitamin D status with fall risk and physical performance presented in our study might also be related to its impact on PTH and OC—a bone turnover marker. We found a significant negative correlation between the above-mentioned bone metabolism markers and 25(OH)D concentration. It has been previously proven that vitamin D deficiency may impair calcium metabolism and increase PTH concentration (Pludowski et al., 2013; Niculescu et al., 2020). A similar relationship was previously presented between vitamin D and OC. A study conducted by Kuchuk and coworkers indicated that in humans with lower vitamin D concentration, a higher concentration of OC is observed (Kuchuk et al., 2009). The concentration of circulating osteocalcin may also be increased in hyperparathyroidism. Previous research showed a significant correlation between OC and PTH (Delmas et al., 1986); however, this correlation was insignificant in our study. Besides that, increased PTH is associated with a higher risk of fractures, bone loss, and osteomalacia (Pludowski et al., 2013). It is also a risk factor for falls and impaired balance among elders (de Franca et al., 2019). Previous studies indicated that elevated PTH concentration is associated with worse performance in the TUG test, lower gait speed, higher risk of sarcopenia, increased instability, and fall risk (Visser et al., 2003; Montero-Odasso et al., 2016). Our results are in line with these observations. A positive correlation between PTH concentration with fall risk and a negative correlation with physical performance measured by TUG and 6MWT has been found. Interestingly, multivariate regression analysis revealed that also OC tends to be a predictor of fall risk. Although no previous studies assessed the association of total OC and fall risk, a study conducted by Vitale J.A. and coworkers indicated that carboxylated osteocalcin is positively correlated with fall risk among older, fractured women (Vitale et al., 2021).

It is well known that in addition to vitamin D insufficiency, another factor associated with impairments in physical performance and higher fall risk is lack of physical activity and a sedentary lifestyle. Several training strategies such as resistance, balance, or tai chi training are recommended for the elderly to reduce the risk of disability and falls (Papa et al., 2017; Hewitt et al., 2018; Penn et al., 2019). The multi-component training programs—including balance, functional, strengthening, and endurance exercises are suggested to be the most effective in improving gait, balance, muscle strength, coordination, and overall physical functioning (El-Khoury et al., 2013). The training procedure applied in the current study also appears successful in improving physical performance. Consequently, it may contribute to healthy aging cause after 12 weeks of moderate-intensity aerobic training combined with resistance exercises, significant improvement in performed functional tests (TUG and 6MWT) and fall risk reduction was observed. Moreover, to the best of our knowledge, no previous studies assess if physical performance improvement after training is related to baseline 25(OH)D concentration. Several studies have assessed whether vitamin D supplementation will enhance the training effect. One study performed on high-level, well-trained athletes indicated that 8 weeks of high-intensity interval training combined with vitamin D supplementation did not induce better training responses than training alone (Jastrzebska et al., 2016). Also, a study by Kirsti Uusi-Rasi and coworkers indicated that both multimodal training and training supported by vitamin D supplementation improved muscle strength, balance, and mobility of older adults. Vitamin D did not enhance exercise effects on physical functioning. Still, some positive effects of supplementation were observed—vitamin D reduced bone loss at the femoral neck and increased trabecular density at the distal tibia (Uusi-Rasi et al., 2015). In the present study, no differences between groups in physical performance improvement and fall risk reduction were pointed out. Both training groups performed better in TUG, 6MWT, and fall risk tests. Therefore, physical training may be the most effective strategy for preventing falls and improving physical performance in older adults. However, there were discrepancies in PTH and OC concentration changes; PTH and OC concentrations were reduced after 12 weeks of training but only in the insufficient vitamin D concentration group. The studies investigating post-exercise changes in PTH concentration are still inconclusive. The review of Lombardi and coworkers indicated that after a single bout of exercise, mainly an increase in PTH secretion was observed, especially after long-lasting and moderate to high-intensity exercises. On the other hand, long-term training may have the opposite effect and limit PTH secretion, particularly among older adults (Lombardi et al., 2020). After 12 weeks of nordic-walking training, a decrease in PTH concentration was observed among postmenopausal women with baseline vitamin D insufficiency (below 18.6 ng/ml) (Nowak et al., 2020), which corresponds to the results obtained in our study. Therefore, this effect may benefit older adults with secondary hyperparathyroidism related to vitamin D deficiency. The impact of aerobic exercise on OC concentration is not fully understood. Both an increase (Kim et al., 2015; Kortas et al., 2020) no changes (Nowak et al., 2020) and a decrease (Wieczorek-Baranowska et al., 2012) was observed. On the one hand, OC is an indicator of bone tissue synthesis, but on the other, its high level reflects intense bone metabolism, leading to increased tissue degradation. A study conducted by Iki and coworkers indicated that subjects at risk of osteoporosis progression, especially the lumbar spine, had significantly higher serum OC concentration (Iki et al., 2006). The current study revealed a decrease in OC concentration, but only in older women with insufficient vitamin D concentration. Among participants with sufficient vitamin D concentration, no changes were observed. It has been previously suggested that reducing the bone turnover rate after training interventions may be favorable for bone mass, especially in people with vitamin D insufficiency (Wieczorek-Baranowska et al., 2012). Nevertheless, future studies are needed to evaluate the effect of exercise on OC concentration and its’ associations with bone metabolism.

Oxidative stress and ROS generation play an important role in aging and age-related diseases—diabetes, neurodegenerative diseases, cardiovascular diseases, sarcopenia, and frailty (Liguori et al., 2018). Physical activity is suggested to contribute to healthy aging by its antioxidant effect (Bachi et al., 2019). In the current study, a significant increase in SH groups concentration in both training groups, which refers to better antioxidant capacity, was observed. There is a lack of studies assessing post-training modifications of the SH groups among older adults; however, studies on young (Metin et al., 2003) and middle-aged participants (de Oliveira et al., 2012), as well as animal studies (Dos Santos et al., 2021), confirmed our observations. It is well known that intensified oxidative stress is associated with a reduced level of serum SH groups, which are components of extracellular antioxidant machinery (Bourgonje et al., 2021). Therefore, regular physical training, particularly aerobic, may elevate antioxidant enzymes content and activity, regardless of age. Among elders, it may also attenuate the effect of oxidative stress on age-related changes (Bachi et al., 2019). Moreover, it has been shown that reduced serum SH groups may be a risk factor for cardiovascular events, type 2 diabetes, and rheumatoid arthritis (Giustarini et al., 2005; Piwowar et al., 2009; Bourgonje et al., 2021). Thus, we are very far from any speculations, but an increase in serum SH groups in our study after 12 weeks of training may have a protective effect on age-related diseases. Unfortunately, this study did not note any significant decrease in MDA concentration as observed in previous studies on older adults after 12 weeks of nordic-walking training (Kortas et al., 2017) and after 16 weeks of combined aerobic-resistance training (Mota et al., 2019).

The study’s limitations should be discussed in the context of the reported results and findings. Firstly, the number of participants was limited. Secondly, the vitamin D supplements were taken by the participants themselves (2,000 units or more per day). Nowadays, the LC-MS/MS HPLC method is the gold standard to measure 25(OH)D; thus, this method should be performed instead of ELISA in future studies. Another limitation is that also other BTM’s such as sclerostin, serum C-terminal telopeptide of type I collagen, or serum procollagen type I N propeptide, as well as bone mineral density, should be investigated further to assess the effect of training and vitamin D status on bone metabolism and bone mass. However, in this research, we focused only on healthy, older women, verification whether observed changes are sex-dependent should be considered in the future. Also, applying this type of training to elders with comorbidities seems essential to investigate whether this type of training acts in the same way as in healthy women.

In conclusion, vitamin D concentration among older women was related to physical performance, fall risk, inflammation, and bone metabolism markers. Consequently, maintaining the optimal vitamin D concentration may be a potential, modifiable factor protecting from declines in physical performance and loss of independence among elders. Moreover, obtained results indicate that 12 weeks of combined training effectively improved elders’ physical performance and increased antioxidant protection, regardless of baseline vitamin D status, indicating the physical activity as the most crucial factor leading to healthy aging. Still, the favorable effect of regular training on bone metabolism tends to be more pronounced in participants with vitamin D insufficiency. Future research is needed to evaluate the role of vitamin D in enhancing physical functioning in humans.

## Data Availability

The raw data supporting the conclusions of this article will be made available by the authors, without undue reservation.

## Abbreviations

PTH, parathormone; BTM, bone turnover markers; OC, osteocalcin; ROS, reactive oxygen species; IL-6, interleukin-6; SH, sulfhydryl groups; MDA, malondialdehyde; 6MWT, 6 min walk test; TUG, time up and go test; FRT, fall risk test; FFM, free fat mass; FM, body fat mass; SMM, skeletal muscle mass; VFA, visceral fat area; SI, stability index.
